# *Haemoproteus* spp. and *Leucocytozoon californicus* Coinfection in a Merlin (*Falco colombarius*)

**DOI:** 10.3390/pathogens9040263

**Published:** 2020-04-04

**Authors:** Simona Nardoni, Francesca Parisi, Guido Rocchigiani, Renato Ceccherelli, Francesca Mancianti, Alessandro Poli

**Affiliations:** 1 Dipartimento di Scienze Veterinarie- Università di Pisa, Viale delle Piagge n. 2, 56124 Pisa, Italy; simona.nardoni@unipi.it (S.N.); guido.rocchigiani.g@gmail.com (G.R.); francesca.mancianti@unipi.it (F.M.); alessandro.poli@unipi.it (A.P.); 2 Centro Recupero Uccelli Marini e Acquatici-CRUMA, via delle Sorgenti 430, 57121 Livorno, Italy; apusvet.cruma@libero.it

**Keywords:** Leucocytozoon californicus, Haemoproteus sp., Falco columbarius, PCR, anatomopathological findings

## Abstract

The *Leucocytozoon* genus comprises numerous widely distributed parasites which have been less investigated than other avian hemoprotozoa. Their occurrence is common, with very variable prevalence values and pathogenicity degrees. *Leucocytozoon* species are characterized by a great taxonomic diversity, and infections are usually restricted to birds of the same family. In the present paper, a mixed hemosporidia infection by *Leucocytozoon californicus* and *Haemoproteus* sp. in an adult male merlin (*Falco columbarius*) which died during hospitalisation is reported, indicating, for the first time, a newly described avian host species. A molecular investigation was carried out through *cytochrome* b gene analysis, revealing a 100% match with *L. californicus* and *Haemoproteus* spp. A blood smear examination allowed us to detect *Leucocytozoon* fusiform mature gametocytes and different degrees of maturity of *Haemoproteus* gametocytes. Histopathology revealed foci of necrosis, hemorrhagic areas and extramedullary hematopoiesis in the liver, the presence of microthrombi in the heart and lung and scattered hemorrhages in the lung.

## 1. Introduction

Avian hemoprotozoa encompass different genera of blood parasites, including *Leucocytozoon*, *Haemoproteus* and *Plasmodium*. These three parasite genera are commonly reported as being pathogenic [1,2]. Almost all parasite species present gametogony and sporogony in blood suckling dipteran vectors, infected through the blood meal. Exoerytrhrocytic schizonts develop in different tissues, while gametocytes occur into the blood cells of infected birds. Nevertheless, developmental stages in the avian host remain only partially studied [3]. Although several parasite species have been encountered from a variety of different countries, information about complete life-cycles of most *Haemoproteus* is lacking, especially for exoerythrocytic stages. In this genus, the sporozoites, inoculated into birds by *Ceratopogonidae* and *Hippoboscidae* Diptera, undergo schizogony in the endothelial cells and probably in fixed macrophages, or myofibroblasts, depending on the parasite species. Gametocytes develop in mature erythrocytes. *Simuliidae* are the cyclic vectors of *Leucocytozoon* parasites that inoculate the sporozoite stages. The exoerythrocytic stages in birds firstly occur in the hepatocytes, in macrophages and other reticuloendothelial cells, then further schizogony stages are observed in the lungs, and less often in the liver, spleen, kidneys, heart, skeletal musculature and other organs. The gametocytes colonize erythroblasts, erythrocytes and mononuclear leukocytes [3].

Among hemoprotozoa, *Plasmodium* spp have the widest host range [4]. Although the majority of *Haemoproteus* and *Leucocytozoon* species are characterised by great taxonomic diversity, in birds, they are relatively species-specific, with infections usually restricted to subjects within the same taxonomic family.

*Haemoproteus* spp are the most common varieties. There are up to 200 species within the genus and they have been reported in 1700 species of birds. *Haemoproteus* parasites have low pathogenicity and infections are usually subclinical; sometimes they cause mild clinical signs and are rarely fatal [1,2].

*Leucocytozoon* appears to be the least investigated among avian haemoprotozoa genera. It is reported especially in Northern temperate areas, where both *Leucocytozoon* and black flies are common [5]. It generally occurs in Anseriformes, turkeys, raptors, wild birds and Columbiformes [6]. *Leucocytozoon* spp are usually considered nonpathogenic in adult raptors, even if they are reported as a possible cause of mortality during reproduction [7] and in nestlings, and as a consequence, need to switch to a new avian host. Pathogenicity would also be correlated with parasite species and parasitemia level [8]. 

Recent studies suggest that some species among avian hemoprotozoa could sometimes be lethal for birds, much more frequently than previously reported [1,2,9]. From this perspective, studies on the distribution and the diversity of avian haemoprotozoa are necessary. These studies would be extremely useful to understand wildlife diseases, particularly the virulence and the mortality rates in avian species due to these parasites. 

The present case report describes, for the first time, a coinfection due to *Leucocytozoon californicus* and *Haemoproteus* sp. in a merlin (*Falco columbarius*) from Italy. 

## 2. Results

### 2.1. Blood Smears

**Figure 1:**
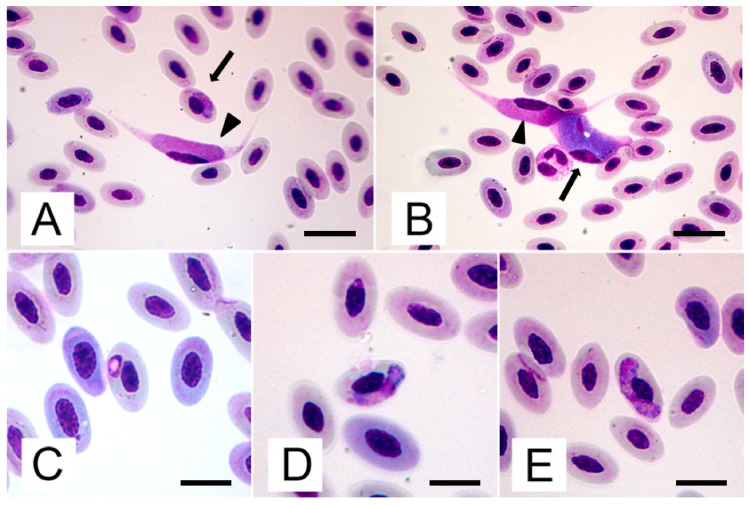
Coinfection of *Haemoproteus* sp. and *Leucocytozoon californicus* from a blood smear of a *Falco colombarius*: **A**) Early stage of gametocytes of *Haemoproteus* sp. in RBC (arrow) and mature microgametocyte of *L. californicus* (arrowhead); **B**) Mature macrogrametocyte (arrow) and microgametocyte (arrowhead) of *L. californicus*; **C**) Ring-shaped young gametocyte of *Haemoproteus* sp in RBC; **D**) Mature microgametocyte of *Haemoproteus* sp. in RBC; **E**) Mature macrogametocyte of *Haemoproteus* sp. in RBC. (Modified Wright’s solution staining; Scale bar A and B 20 µm, C–E 15 µm).

An examination of blood smears revealed the presence of different intracellular stages of parasites consistent with *Haemoproteus* sp. and *Leucocytozoon* sp. (Figure 1A). *Haemoproteus* sp gametocytes were found in RBCs, while it was difficult to establish the host cells for *Leucocytozoon*, since the parasite dramatically changes the morphology of infected cells and no young stages compatible with *Leucocytozoon* have been successfully identified in blood cells. *Leucocytozoon* sp.-infected cells per 1000 blood cells were 10 ± 4, while *Haemoproteus* sp.-infected RBCs per 1000 cells were 66 ± 6. Mature gametocytes were the only clearly visible parasite stage. They filled up the whole cellular space, replacing cell cytoplasm, which sometimes formed elongated “horns” (Figure 1A,B). The measurements of mature gametocytes are presented in Table 1. Microgametocytes stained more lightly than the macrogametocytes. The cytoplasm was extremely pale blue and the nucleus was pale pink. Macrogametocytes could be distinguished from microgametocytes because of their darker appearance, i.e., cytoplasm stained dark blue and nucleus light red, and small vacuoles and magenta volutin cytoplasmatic granules could be frequently seen (Figure 1B). The nucleus of macrogametocytes was also smaller than the that of microgametocytes.

Immature and mature gametocytes of *Haemoproteus* sp were visible in RBCs. Young gametocytes (Figure 1C) were roundish to oval with an even outline, a peripheral dark nucleus and a large white vacuole. They grew toward the host cell nucleus and had no contact with the host cell membrane or nucleus. Very small, scattered pigment granules of hemozoin could be seen at low magnification. Ring gametocytes developed in length and width and evolved in medium grown gametocytes of elongated shape with a wavy outline. At this stage, they did not fill the erythrocytes up to their poles; they were appressed to the nucleus, but rarely in contact with the envelope of the host cells, and irregular to roundish small pigment granules could be seen (Figure 1A). Mature gametocytes could be easily differentiated both in macro- and micro- gametocytes. Macrogametocytes (Figure 1E) were sausage-shaped, with an angular outline. The mature stages filled the erythrocytes up to their poles, although not completely encircling the nucleus, they were closely appressed to it and rarely appeared to be in contact with the envelope of the host cells, with some variations. The cytoplasm was moderately coarse and stained pink, with few small, scattered malaria-like pigment granules. Granules of volutin could also occur. Vacuoles regressed, but sometimes a small vacuolar residue could be observed. The nucleus was terminal or subterminal, far from the host cell nucleus, and stained bright pink (average length 11.5 ± 1.5 and width 2.6 ± 0.4).

The general development and configuration of microgametocytes are similar to the macrogametocytes, albeit with a slightly defined outline. The cytoplasm was finely granular and stained pink, with medium size malaria and valutin pigment granules accumulated on the membrane of the polar regions. The vacuole residue could be seen. Nucleus was median or submedian, close to the host cell nucleus, with some variations, and stained bright pink. 

### 2.2. Molecular Analysis

**Figure 2:**
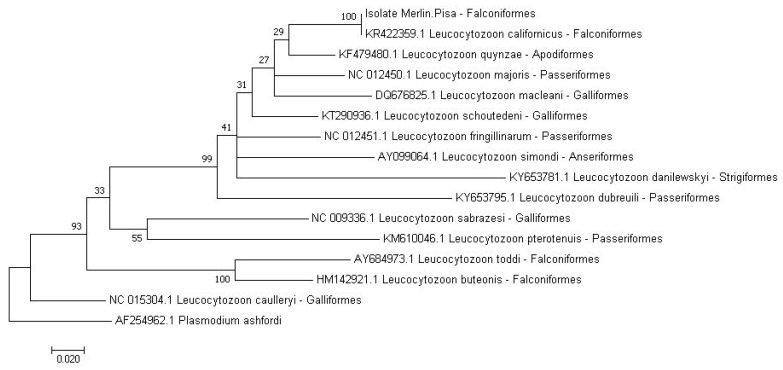
Phylogenetic tree showing the *Leucocytozoon* sequencing results.

Nested-PCR was positive for *Leucocytozoon* and *Haemoproteus/Plasmodium* DNA. The nucleotide sequence of the isolates (493 bp and 417 bp, respectively) showed a 100% identity with *Leucocytozoon californicus* (Figure 2) [GenBank accession number: KR422359.1] and with *Haemoproteus* sp. H-FATI1 [GenBank: EF607289.1], isolated from a *Falco tinnunculus* by Krone et al. [10], who provided a detailed phylogenetic tree.

### 2.3. Pathological Investigations

Necropsy revealed poor body conditions with a reduction of muscle mass and fat deposits. The proventriculus was empty and the only gross abnormality findings observed during the necropsy were severe hepatomegaly and scattered hemorrhages in the liver and the lung.

**Figure 3:**
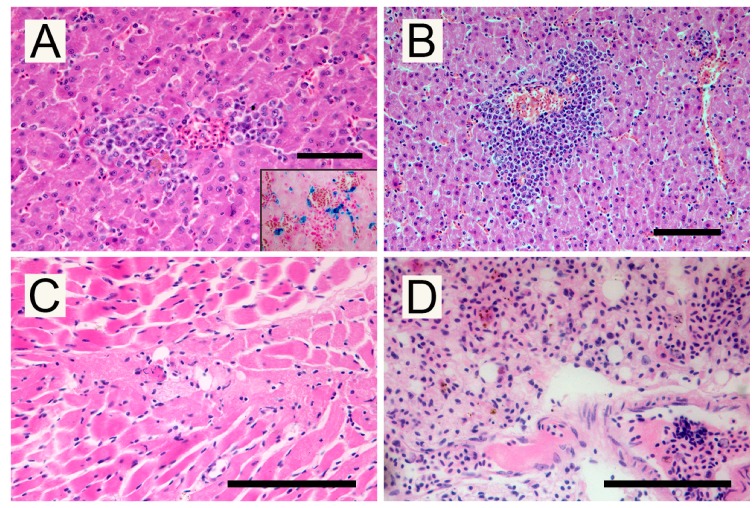
Hematoxylin and eosin-stained tissue from a *Falco colombarius*: (**A**) Foci of necrosis and hemorrhages in the liver; insert: Perl’s stain, macrophages laden with hemosiderin; (**B**) Myeloid precursors in extramedullary hematopoiesis in the liver; (**C**) Thrombus in a cardiac capillary with necrosis of the surrounding tissue; (**D**) Thrombi in pulmonary vessels. (H–E; Scale bar 100 µm).

Histologically, tissue stages of *Leucocytozoon*, such as macromeronts and micromeronts, were not detected. However, histopathology investigations allowed us to observe tissue changes, probably related to the colonization of host tissues (Figure 3). 

In the liver, small necrotic foci were surrounded by scattered Perl’s-positive hemosiderin-laden macrophages, which are interpreted as posthemorrhagic lesions (Figure 3A). Multifocal aggregates of myeloid precursors cells, interpreted as foci of extramedullary hematopoiesis, were observed with a perivascular arrangement (Figure 3B). Hepatic sinusoids were dilated due to blood congestion. Mature stages of the parasite were evident within the thrombi in medium and small vessels of examined organs. Necrosis of miocardiocytes and microthrombi in small vessels (Figure 3C) were detected in the heart. In the lung, the gametocytes formed clusters together with red blood cells and oxyphilic filaments of fibrin, obliterating the lumina of alveolar capillaries vessels (Figure 3D). 

## 3. Discussion

The present report describes, for the first time, a parasite coinfection of *Haemoproteus* sp. and *L. californicus* in *F. colombarius,* a widely distributed diurnal raptor. Based on our findings, this avian species could be an additional host for *L. californicus.*

Gametocytes of *Haemoproteus* sp. developed in RBCs, while it was not easy to identify the host cell for *L. californicus,* since the parasite dramatically modifies the morphology of infected cells. In fact, even if the parasite is reported to infect blood cells, it seems that *Leucocytozoon* spp. would usually infect cells with a single nucleus. Therefore these protozoa could infect RBCs (since avian RBCs are nucleated), single nucleus WBCs, or single nucleated thrombocytes (another characteristic feature of avian blood [11]. Moreover, we could not identify any possible immature stage of *L. californicus*, since the only immature stage we found was characterised by the presence of pigment granules, which are known to be absent in *Leucocytozoon* spp. [3]. On the other hand, mature gametocytes in the blood smear were easily be ascribable to *L. californicus* or *Haemoproteus* sp., since the *L. californicus* host cell–parasite complex was characterised by parasitic forms developing in a fusiform structure, as reported in other cases of infection of diurnal raptors [12,13]. This was very different to the sausage-shaped mature gametocytes of *Haemoproteus* spp. *Leucocytozoon* gametocytes, whose size was comparable to that described in kestrel by Sacchi and Prigioni [14]. However, parasite width and area agreed with the description reported by Walther et al. [15]. 

Numerous gametocytes were microscopically detectable in blood smears, suggesting a massive presence in the host’s body. This was probably due to the high individual susceptibility of the host, suffering the consequences of both physical injury and hospitalization. Such events could have played a role in impairing the immune system, leading to death, probably caused by hemosporidia infection, as supported by both clinical and histological evidence. 

Pathological findings suggest that the hemosporidian parasite infection could have been fatal for the subject. Hematological changes, probably related to the high parasitaemia, could have led to the formation of thrombi exiting in capillary obstructions. Moreover, the death resulted from cardio-respiratory failure, caused by clots due to subsequent disseminated intravascular coagulation. 

Parasitic tissue stages were not detected in samples from organs, while indirect signs of parasite tissue colonization, such as necrosis and hemorrhages, were observed, in agreement with Valkiūnas [3] and Donovan et al. [2]. Moreover, extramedullary hematopoiesis represents a typical finding in avian malarial disease [16,17], probably due to a compensation mechanism of severe anemia, the most common clinical finding in erythrocytic stages of the parasites.

The apicomplexan *cytochrome* b gene is genetically informative, and it has been proven to be suitable to investigate the evolution and divergence of avian malarial parasites in different host groups, to analyze the areas of parasite transmission [18], and to carry out phylogenetic studies for *Leucocytozoon* species [19]. 

## 4. Materials and Methods

### 4.1. Case History

A male adult *F. colombarius* was hospitalized as a consequence of an injury to the flight feathers due to a gunshot wound, at the Centro Recupero Uccelli Marini ed Acquatici-CRUMA, a wild bird care center in Livorno (Italy). Despite a satisfactory clinical response and wound remission, a sudden clinical worsening with respiratory distress arose, leading to the quick death of the bird.

### 4.2. Blood Smears

Blood in ethylenediaminetetraacetic acid (EDTA) was collected before death. Smears were prepared on a glass slide, fixed in methanol and stained with modified Wright’s solution. Each smear was examined through 100–150 fields at low magnification (400X) and then at least 100 fields at high magnification (1000X). The measurements of parasite and infected host cells were made based on morphometric parameters of gametocytes as previously proposed, i.e., the length, width, and area of macrogametocytes and microgametocytes, parasite nucleus, and area of host cell parasite complex [20]. The parameters were determined using a semiautomated morphometric system (LAS 4.1, Leica Microsystems, Wetzlar, Germany) and statistically analysed using IBM SPSS Statistics 21 system. 

### 4.3. Molecular Analysis

Part of blood was stored at 4 °C for molecular analysis. DNA was extracted using the blood/cultured cell genomic DNA extraction mini kit (Fisher Molecular Biology, Roma, Italy), following the manufacturer recommendations. Hemoprotozoa DNA was detected with a nested PCR protocol targeting the cytochrome b mitochondrial gene, according to Hellgreen et al. [19] Primers for first step amplification were HaemNFI (5’-CATATATTAAGAGAAITATGGAG- 3’) and HaemNR3 (5’-ATAGAAAGATAAGAAATACCATTC-3’). Two second step PCR were then performed, both using the product of the first step as a template. HaemF (5’-ATGGTGTTTTAGATACTTACATT-3’) and HaemR2 (5’-GCATTATCTGGATGTGATAATGGT-3’) internal primers were used for detection of *Haemoproteus* and *Plasmodium* [21] amplifying a 478 bp sequence. *Leucocytozoon* lineages were amplified by HaemFL (5’-ATGGTGTTTTAGATACTTACATT-3’) and HaemR2L (5’-CATTATCTGGATGAGATAATGGIGC-3) amplifying a 480 bp sequence. All PCRs were carried out in a total volume of 40 µL, using 6 and 3 µL of template DNA for the first and the second reaction, respectively, 8 µL of reaction buffer, 1 mM of each primer, 1.25 U of wonder Taq DNA polymerase (Euroclone, Milan, Italy), and 24 and 27 µL of nuclease-free water for the first and the second reaction. Thermal cycling was performed in a C1000 thermal cycler (Bio-Rad, Hercules, CA, USA) using the following conditions: 30 s at 94 °C, 30 s at 50 °C and 45 s at 72 °C for 20 cycles for the first reaction and for 35 cycles for the second. Positive and negative samples were added for quality control. Amplified DNA products, as well as molecular weight markers (Sharp Mass TM 100 Plus Ladder, Euroclone, Milano, Italy), were subsequently submitted to electrophoresis in 2% agarose gel at 100 V for 30 min. The gel was prestained with EuroSafe -Fluorescent Nucleic Acid (Euroclone, Milano, Italy). 

The PCR products were then sequenced by a commercial laboratory (BMR Genomics, Padova, Italy). The sequences were compared to those already deposited in the NCBI database using the BLAST search engine.

### 4.4. Pathological Investigations

Necropsy was carried out and representative tissue samples were collected from heart, lung, liver, spleen, kidney, proventriculus, intestine, and central nervous system. Samples were fixed in 10% buffered formalin (pH 7.4), routinely processed and embedded in paraffin. Four-micron thick sections were stained with hematoxylin and eosin for general vision and specific stain (Periodic Acid Schiff—PAS) for fungi, acid-fast bacteria (Ziehl Neelsen stain) and iron-loaded hemosiderin pigment (Perl’s stain).

## 5. Conclusions

The present study reported, for the first time, the occurrence of *L. californicus* in coinfection with *Haemoproteus* sp. in *F. colombarius*. The concomitance between the detection of RBC stages of parasites and the lack of identification of tissue forms in the sampled organs, together with pathological signs attributable to pre-erythrocytic stages and extramedullary hematopoiesis, highlights the fatal outcome. 

## Figures and Tables

**Table 1:** Measurements of mature gametocytes and cell-parasite complex of *Leucocytozoon californicus* from blood smears of the examined *Falco columbarius*.

Figure .		Macrogametocytes (n=10)	Microgametocytes (n=10)
		Min – Max (Mean ± SD) µm	Min – Max (Mean ± SD) µm
	Lenght	19.0 – 21.6 (20.5 ± 0.7)	18.0 – 20.9 (19.4 ± 1.2)
**Parasite**	Width	8.2 – 10.2 (9.1 ± 0.9)	5.8 – 7.2 (6.5 ± 0.6)
	Area	87.0 – 147.8 (123.9 ± 24.4)	69.1 – 98.0 (83.7 ± 11.4)
	Lenght	3.1 – 4.8 (4.1 ± 0.7)	6.9 – 8.9 (7.7 ± 0.8)
**Parasite nucleus**	Width	2.0 – 2.9 (2.5 ± 0.4)	3.3 – 5.4 (4.2 ± 0.9)
	Area	6.7 – 11.7 (8.8 ± 2.0)	20.8 – 32.0 (27.2 ± 4.4)
**Cell-parasite complex**	Area	128.7 – 230.0 (189.6 ± 40.7)	130.0 – 197.50 (163.2 ±25.6)
